# The establishment and biomechanical analysis of finite element model for halo-pelvic traction in scoliosis correction

**DOI:** 10.3389/fphys.2025.1670376

**Published:** 2025-11-13

**Authors:** Wanzhong Yang, Wei Guo, Jie Yang, Honglai Zhang, Zemin Wang, Shiyong Wang, Jianqun Zhang, Xiaoyin Liu, Rong Ma, Zhaohui Ge

**Affiliations:** 1 Department of Orthopedic, General Hospital of Ningxia Medical University, Yinchuan, China; 2 The First Clinical Medical College, Ningxia Medical University, Yinchuan, China

**Keywords:** halo-pelvic traction, scoliosis, finite element model, model validation, biomechanical analysis

## Abstract

**Background:**

Halo-pelvic traction (HPT) is increasingly used for severe spinal deformity correction, but its biomechanical mechanisms remain poorly understood, particularly lacking of a comprehensive finite element model incorporating the complete Halo-pelvic-spine-pelvis construct. This study aimed to establish a comprehensive halo-pelvic traction model and assess its biomechanical reliability.

**Methods:**

A severe kyphoscoliosis (SS) model was created using patient CT data, reconstructed in Mimics, optimized in Geomagic Wrap, and assembled in UG12 for finite element analysis in Ansys. Material properties and appropriate boundary conditions were defined. Model validity was verified by measuring geometric parameters and the stress loading tests including T1-T4 range of motion, T12-L2 stiffness and L4-L5 displacement and comparing results with published data. An equivalent adolescent idiopathic scoliosis model was also developed (MS). Both models were analyzed under traction displacements (50, 80, 100, 125 and 150 mm).

**Results:**

The SS model closely matched clinical measurements with differences of less than 0.5° in Cobb and less than 2 mm in apical vertebral translation and spinal balance parameters. The T1-T4 mobility was lower than literature values, due to the involvement of T1-T4 segments in compensatory curvature formation. The T12-L2 stiffness and L4-5 displacement were consistent with published data, confirming the model’s validity. Using patient weight-based maximum traction force, the MS model results demonstrated that at 150 mm distraction distance, the pelvic pins reached their maximum von Mises stress of 956.99 MPa with 2.29% pelvic pin tract strain, while at 125 mm, the cranial pins showed 2.39% strain and the support rod reaction force exceeded the maximum traction force. In the SS model, as traction increased, the stresses in both pelvic and cranial pins, pelvic pin tract strain, and support rod reaction forces all showed increasing trends, peaking at 150 mm without reaching critical thresholds. The cranial pin tract strain followed the same trend, reaching 2.26% at 150 mm.

**Conclusion:**

The validated finite element models demonstrate high anatomical and biomechanical accuracy. HPT may serve as an interim treatment for MS models with strict traction force and displacement control. While rigid SS models require dynamic force adjustment and distraction distances maintained below 150 mm for optimal safety.

## 1 Introduction

Scoliosis is a complex three-dimensional spinal deformity. In severe cases, it is accompanied by significant cardiopulmonary impairment. The continuous progression of the deformity can lead to spinal rigidity, which not only affects the appearance but also impacts the patient’s mental health, severely diminishing the quality of life (Sucato, 2010; Gollogly et al., 2004). For severe spinal deformities, although osteotomy alone can achieve satisfactory corrective results and restore spinal balance, its drawbacks include prolonged operative time, significant blood loss, high osteotomy levels, and an increased risk of postoperative complications (Papadopoulos et al., 2015; Riley et al., 2018). Longitudinal spinal traction can enhance the flexibility of the spine, significantly increase the volume of the thoracic and abdominal cavities, markedly and effectively improve the patient’s cardiopulmonary function, elevate the patient’s nutritional status, reduce the risk of secondary corrective surgery, and increase the rate of deformity correction (Nemani et al., 2015; Chan et al., 2016; Wang et al., 2021). The halo pelvic traction provides continuous and controllable corrective traction by gradually extending the support rod through the rotation of the support bar nut (Zeng et al., 2015). Longitudinal traction of the spine causes muscle fatigue, leading to displacement and rupture of tendons, ligaments, blood vessels, and spinal cord cells. The spontaneous repair of tissue cells adapts to a new equilibrium, thereby achieving orthopedic effects (Cobanoglu et al., 2018; LaMont et al., 2019). A recent meta-analysis has demonstrated that preoperative halo-pelvic traction for orthopedic surgery not only achieves satisfactory deformity correction but also significantly improves pulmonary function, suggesting it may be the safest and most effective optimal choice for treating severe scoliosis (Sun et al., 2023).

Halo-pelvic traction offers advantages such as continuous and gradual traction force and a relatively safe traction process. It can circumvent the limitations of skull-femoral traction techniques, including limited traction force, low correction rates, complications like joint stiffness and pressure sores due to prolonged bed rest, as well as the shortcomings of gravity traction, such as restricted traction force and duration, insufficient stability, and poor controllability (Qiu et al., 2007; Pourtaheri et al., 2016). However, the halo-pelvic traction treatment for scoliosis still faces some challenges, including loosening and dislodgement of halo pins and pelvic pins, as well as pin tract fractures (Talamonti et al., 2019; Huang et al., 2023). Many modified halo-pelvic traction techniques have been reported in clinical applications and have demonstrated favorable orthopedic results (Qi et al., 2020; Ilyas et al., 2021). However, the choice of traction force and volume is mostly based on the operator’s clinical experience and the patient’s tolerance level, and this choice is blinded. The appropriate amount of traction has an important influence on the efficacy and complications. Too little traction may lead to poor traction effect, prolonged treatment period and increased risk of nail tract infection. While too much traction may lead to nail tract fracture and nerve injury. In recent years, some scholars have attempted to simulate the orthopedic effects of traction in scoliosis by modeling scoliosis and applying additional forces at the vertebral body, with attention to the changes in traction force and amount of traction, and some progress has been achieved (Pei et al., 2022; Li et al., 2024). However, there are fewer studies on the mechanics of halo-pelvic traction in scoliosis orthopedics, especially there is a lack of a complete finite element model of halo pelvic traction for spinal deformity. Therefore, we propose to construct a finite element model of halo pelvic traction for spinal deformity in this study and simulate and analyze the mechanical changes of halo pelvic traction in the treatment of scoliosis under different amounts of traction, so as to provide a mechanical basis for the use of halo pelvic traction-assisted osteotomy in the clinical treatment plan.

## 2 Materials and methods

### 2.1 Model extraction & optimization

The study was reviewed and approved for consent by the Research Ethics Committee of the General Hospital of Ningxia Medical University (No. KYLL-2024-0590), and the subject patients signed an informed consent form. Siemens Somatom Sensation 64-slice spiral CT was provided by the Department of Radiology at the General Hospital of Ningxia Medical University. Scan data: slice thickness 0.625 mm, image matrix set at 512 × 512. Computer and processing software information: computer configuration: Windows 10 × 64-bit operating system, Intel(R) Core(TM) i7-3700 processor, 1T solid-state drive, 32G memory. A 14-year-old patient with severe lateral kyphosis deformity of the spine, with a height of 138 cm and a weight of 42 kg was selected (Severe Scoliosis Model, SS Model), and the patient’s whole spine CT data were imported into the MIMICS 20.0 (Materalise, Inc., Leuven, Belgium) software in the DICOM format, and the images were subjected to a thresholding segmentation process for the extraction of the whole spine skeletal model. Individual vertebrae and skull were exported in STL format separately. Subsequently, the STL files were imported into Geomagic 2021 (Geomagic, Inc., United States) software for surface optimization, defect repair, drawing surface slices, constructing grids, and fitting surfaces. The initial model was saved in STP file format and imported into UG 12.0 for model assembly.

**FIGURE 1:**
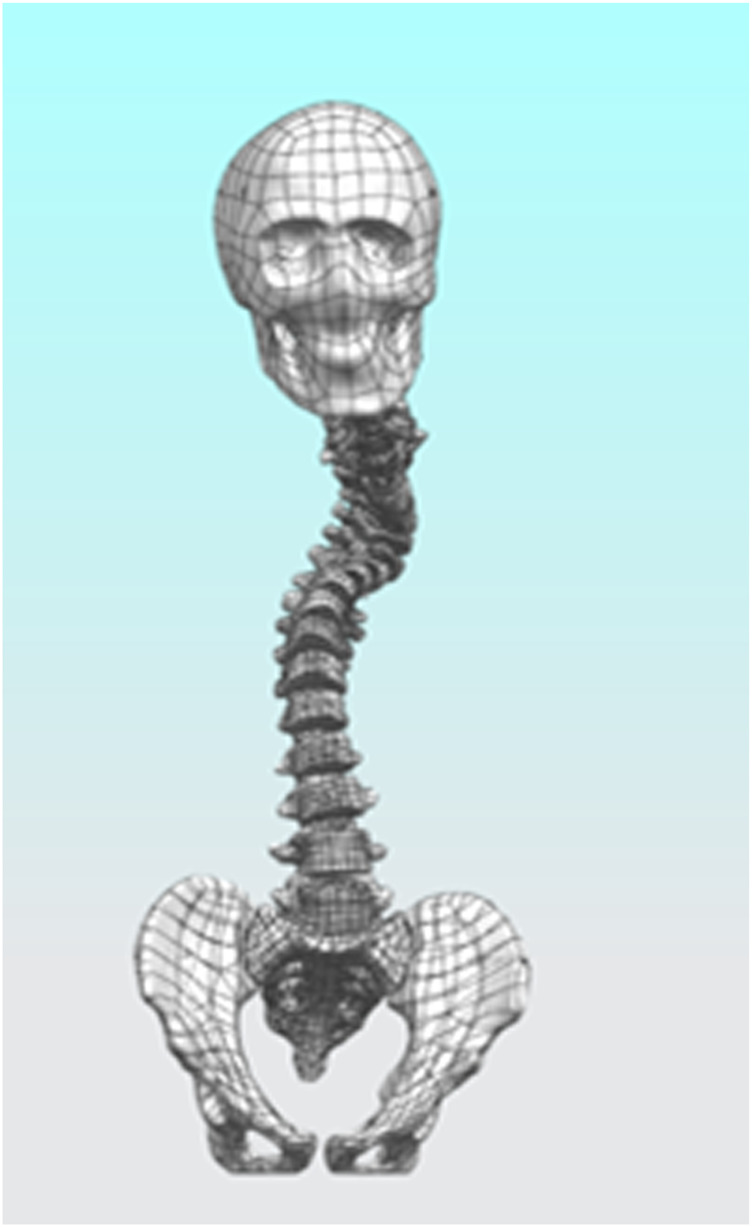
Initial finite element model.

After completing the modeling of each vertebral segment, we proceeded with the construction of the intervertebral disc structure. Using L2 as an example, the vertebra was first isolated, and three points were selected on its superior surface to establish “Datum Plane 1.” On this plane, the “Spline” tool was used to sketch the initial two-dimensional outline of the intervertebral disc, following the contour of the vertebral superior endplate. After exiting the sketch, the “Extruded Boss” feature was applied, with the extrusion set to “Symmetric on Both Sides” and “Do Not Merge Results.” The extrusion height was adjusted to ensure close contact with the endplates of the adjacent superior and inferior vertebrae, forming a preliminary three-dimensional geometry of the intervertebral disc. Subsequently, the extruded body was temporarily hidden, and the “Offset Surface” function was employed to generate zero-distance offset surfaces from the inferior endplate of L1 and the superior endplate of L2. These surfaces were then used as splitting tools to trim the excess portions of the extruded body, resulting in a well-fitted intervertebral disc prototype. Furthermore, based on the same endplate surfaces, additional offset surfaces were created with a 1 mm inward offset. A second segmentation was performed using these surfaces, dividing the intervertebral disc structure into three distinct layers: the central disc body, the superior endplate cartilage, and the inferior endplate cartilage. Considering the anatomical composition of the intervertebral disc, which includes the nucleus pulposus and annulus fibrosus, “Datum Plane 2” was created on the disc model. A contour representing the nucleus pulposus was drawn according to typical anatomical morphology—occupying approximately 50%–60% of the total disc volume—and the “Split” operation was used to separate it from the main disc body. This process ultimately yielded two independent entities: the annulus fibrosus and the nucleus pulposus. The above procedure was repeated to sequentially construct the composite structures of all intervertebral discs (Figure 1).

### 2.2 Constructing a model for halo pelvic traction

**FIGURE 2:**
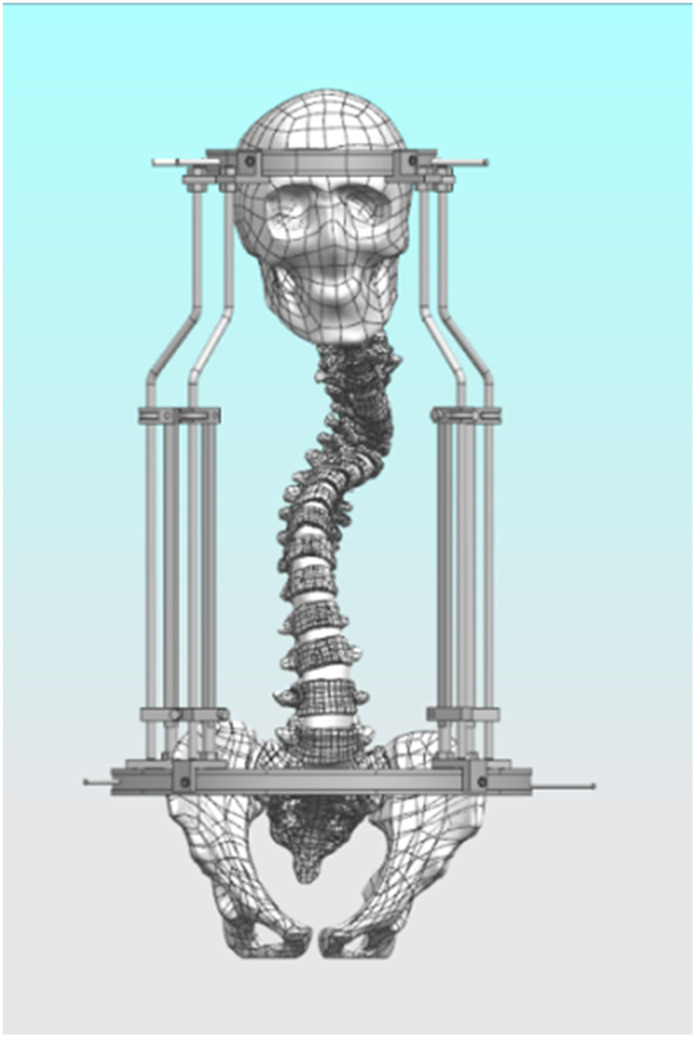
Scoliosis model with assembled halo-pelvic traction device.

**FIGURE 3:**
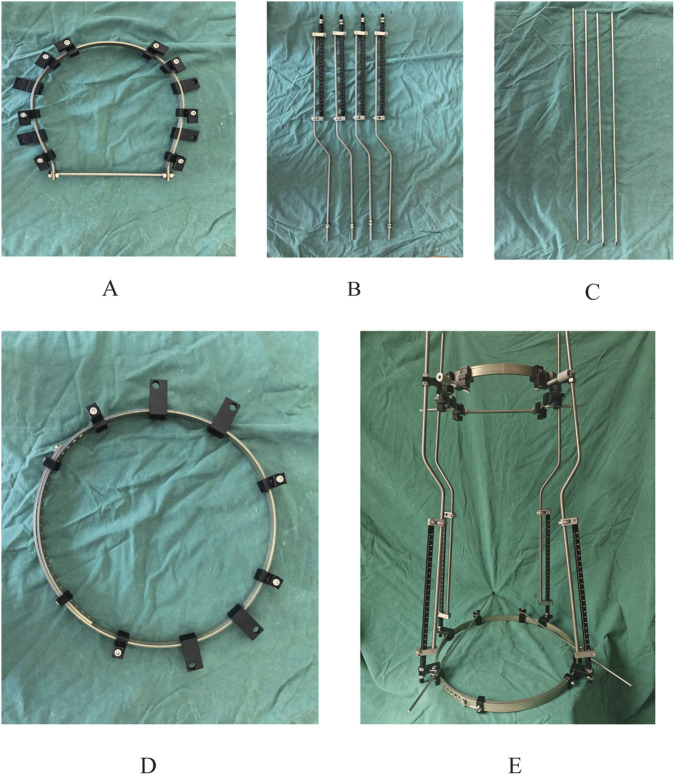
Physical prototype of the halo-pelvic traction device. **(A)** Cranial ring; **(B)** Support rod; **(C)** Cranial and Pelvic pin; **(D)** Pelvic ring; **(E)** Complete halo-pelvic traction frame.

The halo pelvic traction device was constructed in UG 12.0 software by scanning, releasing, stretching, and resection functions. Subsequently, the model was straightened using the functions of rotation and movement in the software, and then the initial model was assembled with the halo-pelvic traction device (Figure 2) and the model was saved in x-t format file. The physical drawing of the halo pelvic traction device is shown in Figure 3.

### 2.3 Material assignment and mesh setup

A static analysis module was created in Ansys 2020 R2 (Ansys, Inc., United States), and the x_t file was imported into this static module. Within the Workbench static analysis environment, a material library was established and populated with reference to literature values (Goel et al., 1995; Polikeit et al., 2003) as shown in Table 1, 2. Distinct material properties were then assigned to each anatomical structure of the model accordingly. Subsequently, the Mechanical window was launched from the Workbench interface. The meshing process was carried out using tetrahedral elements, with careful consideration given to the number of actual model features. Since excessive mesh density would increase computational load, while overly coarse or poor-quality mesh would compromise result accuracy, the mesh settings were fine-tuned based on prior experience to improve both mesh quality and subsequent data accuracy. The following mesh sizes were implemented: 2.0 mm for vertebrae and the halo pelvic ring, 1.0 mm for bone pins, and 0.5 mm for the cranial and pelvic regions in contact with the bone pins. Additionally, key spinal ligaments—including the anterior longitudinal ligament, posterior longitudinal ligament, supraspinous ligament, interspinous ligament, ligamentum flavum, and intertransverse ligament—were modeled using “Spring” elements positioned at their corresponding anatomical locations.

**TABLE 1:** Material properties of each structure in the finite element analysis models.

Materials	Young’s modulus (Mpa)	Poisson ratio	Crosssectional area (mm2)	Reference
Capsular ligament	20	0.3	40	Polikeit et al. (2003)
Anterior longitudinal ligament	20	0.3	38	Polikeit et al. (2003)
Posterior longitudinal ligament	70	0.3	20	Polikeit et al. (2003)
Ligamentum flavum	50	0.3	60	Polikeit et al. (2003)
Supraspinous ligament	28	0.3	35.5	Polikeit et al. (2003)
Interspinous ligament	28	0.3	35.5	Polikeit et al. (2003)
Intertransverse ligament	50	0.3	10	Polikeit et al. (2003)
Cortical bone	12,000	0.3	/	Polikeit et al. (2003)
Cancellous bone	500	0.2	/	Polikeit et al. (2003)
Nucleus pulposus	2	0.499	/	Goel et al. (1995)
Annulus fibrosis	4.2	0.3	/	Goel et al. (1995)

**TABLE 2:** Material properties of 316L Stainless Steel.

Materials	Young’s modulus (Mpa)	Poisson ratio	Tensile strength (Mpa)	Yield strength (Mpa)	Reference
316L (supports and connectors)	187,500	0.3	490	190	MatWeb (2025)
316L (bone pin)	200,000	0.3	860	690	Ren et al. (2019), MatWeb (2025)

### 2.4 Boundary and loading conditions

**FIGURE 4:**
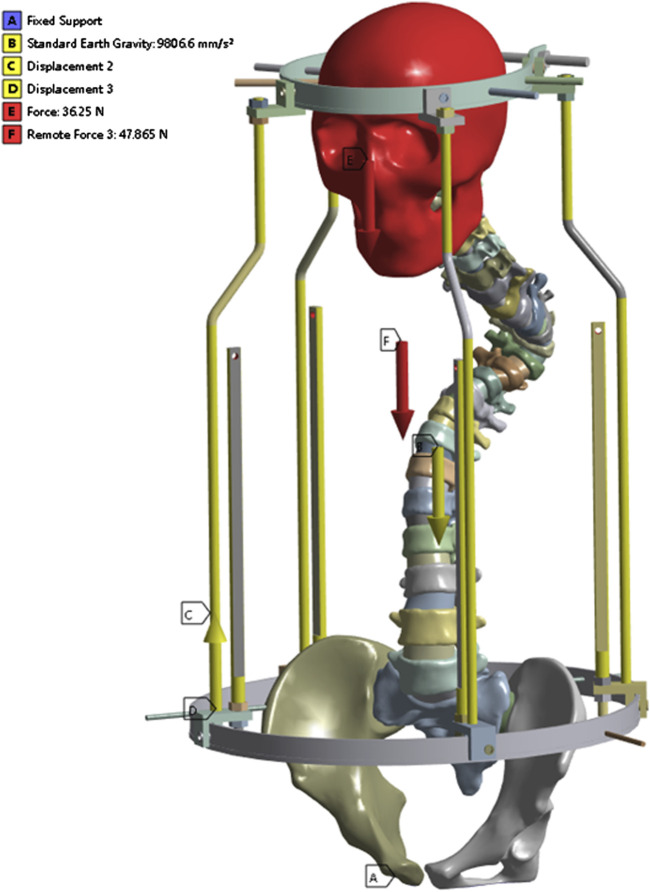
Stress loading and boundary conditions of the halo-pelvic traction device.

The lower surface of the pelvis was set as a fixed constraint, adding gravity of 9,806.6 mm/s2, the brace bar constrained xy direction displacement, and the head-pelvic ring brace bar displacement upward 47 mm. It is known that the T1-L5 cone load is about 50.8% of the body mass as a percentage of the body mass from the literature (Clin et al., 2011), the patient’s mass is about 42 kg and the cranial bone is added with a load of 230 N, taking into account the additional cervical vertebrae. Since this bracket is threaded connection, the halo pelvic ring and pelvic ring are braced by turning the screws, so as long as the force on the cranial ring support rods and pelvic ring support rods are kept the same, the threaded connection can be ignored to keep only the support rods, and according to the previous calculations, the pelvic ring support rods are added with the downward centralized force of 252.85 N. Bone pins and pin channels are friction, and the coefficient of friction is 0.3, and the contact relationship between the other structures is set to be “Boned” (Figure 4).

### 2.5 Model validation

In this study, the validity of the model was verified by geometric morphometric measurements and stress loading tests. Specifically, the geometrical measurements were made by measuring the Cobb angle and vertebral offset in the model and X-ray films. The stress loading tests included three parts: (1) T1-T4 mobility: the degrees of freedom of the lower surface of T4 were restricted in six directions, and a moment of 4 N.m was uniformly applied on the upper surface of T1 in different directions to simulate the flexion-extension, lateral flexion, and rotational working conditions, and the mobility in the different conditions was calculated to compare with the results of the BUSSCHER. (2) Calculating the stiffness of the T12-L2 model: firstly, a vertical compression force of 800 N is uniformly loaded on the upper surface of T12 to calculate the average stiffness of the model. Secondly, a moment of 16,000 N.mm is applied to the upper surface of T12 to observe the angular changes of the segments and the stiffness values during forward flexion, extension, lateral flexion and ratation. (3) L4-5 displacement: Constraining the degree of freedom of the lower surface of the L5 vertebra, loads of 500 N, 1000 N, 1500 N 2000 N were applied to the upper surface of the L4 vertebra, and the simulation results were compared with the literature. A consistent millimeter-based unit system was adopted throughout the finite element analysis: length in mm, force in N, moment in N·mm, and stress in MPa.

### 2.6 MS model establishment

A finite element mode of a 15-year-old patient with idiopathic scoliosis weighing 45 kg and height of 145 cm was established according to the above method and the pelvic ring support rods are added with the downward centralized force of 291.17 N. We defined this model as the minor curve model (MS model).

### 2.7 Measurement parameters at different stretching distances

The displacements, cranial and pelvic maximal equivalent forces, cranial and pelvic pin tract maximal equivalent strains, and halo-pelvic ring support bar reaction forces of the two finite element models were measured at different stretching distances.

## 3 Results

### 3.1 Validation results (SS model)

#### 3.1.1 Geometric model measurement

**FIGURE 5:**
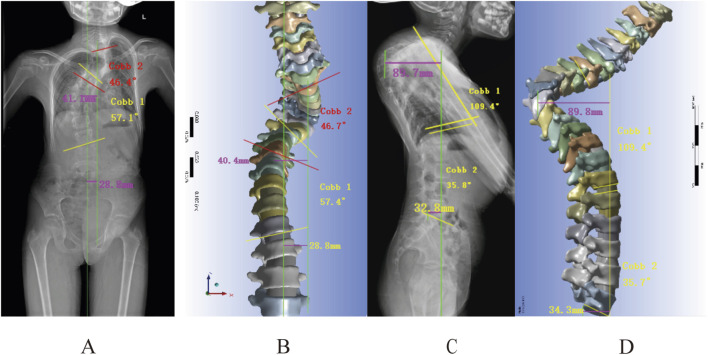
Geometric measurements versus radiographic findings: **(A,B)** Frontal radiograph measurements: Cobb angle, apical vertebral translation, and coronal balance parameters; **(C,D)** Lateral radiograph measurements of kyphotic Cobb, lumbar lordosis, and sagittal vertical axis (SVA).

The radiographs revealed a Cobb of 46.4°for the upper thoracic curve, 57.1° for the main curve, a C7PL-CSV1 of 28.8 mm, and an apical vertebral translation (AVT) of 41.1 mm. The finite element model showed corresponding measurements of 46.7° for the upper thoracic curve, 57.4° for the main curve, a C7PL-CSV1 of 28.8 mm, and an AVT of 40.4 mm, with differences of 0.3°, 0.3°, 0 mm, and 0.7 mm respectively (Figures 5A,B). Lateral radiographs demonstrated a kyphotic Cobb of 110.7°, an L1-L5 Cobb angle of 35.8°, sagittal AVT distance of 89.7 mm and sagittal vertical axis (SVA) of 32.8 mm. The finite element model displayed a kyphotic Cobb of 109.4°, an L1-L5 Cobb angle of 35.7°, a sagittal AVT of 89.8 mm and SVA of 34.3 mm, with differences of 1.3°, 0.1°, 0.1 mm and 1.5 mm, respectively (Figures 5C,D). These results validate the model’s accuracy.

#### 3.1.2 T1-T4 and T12-L2 mobility tests

**FIGURE 6:**
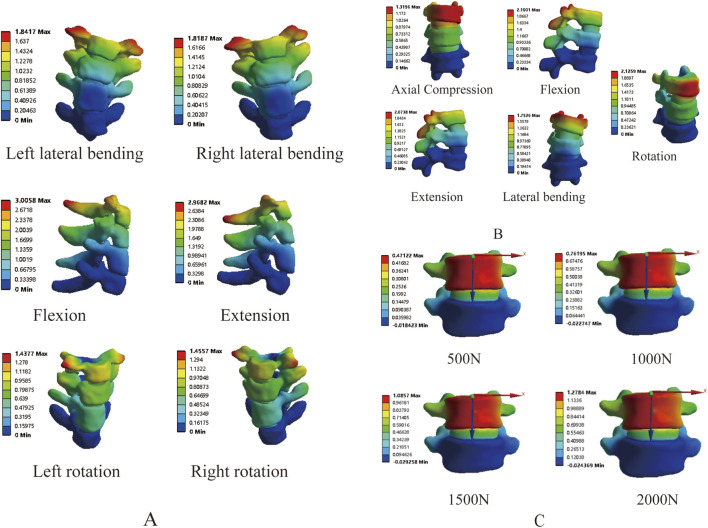
Validation of stress loading protocol: **(A)** T1-T4 range of motion; **(B)** T12-L2 stiffness and mobility; **(C)** L4-5 displacement under vertical loading.

In order to further prove the validity of the model, we measured the mobility of T1-T4 and T12-L2 segements respectively, and the results showed that the mobility of T1-T4 in flexion-extension, lateral flexion and rotation conditions were 2.75°, 4.09° and 4.51°, respectively (Figure 6A), which were lower than the results of the studies of Xin Dach (Xin, 2017) and Busscher (Busscher et al., 2009), which may be due to the fact that, T1-T4 were involved in the composition of the primary bending and thus less active in the present study’s model. The stiffnesses of T12-L2 in axial compression, flexion, extension, lateral flexion, and rotation were 6060 N mm, 1760 N mm, 1740 N mm, 2290 N mm, and 3130 N mm respectively (Figure 6B), which were similar to the results reported in the literature (Markolf, 1972; Panjabi et al., 1977; Tencer et al., 1982).

#### 3.1.3 L4-5 displacement

By applying loads of 500 N, 1000 N, 1500 N, and 2000 N on the superior surface of L4, the results showed displacements of 0.47 mm, 0.76 mm, 1.09 mm, and 1.28 mm, respectively (Figure 6C), which fall within the range reported in the literature (Brown et al., 1957; Markolf, 1972). The above results fully demonstrate the effectiveness of the model, making it suitable for further research.

### 3.2 Preliminary study on traction in the MS model

**FIGURE 7:**
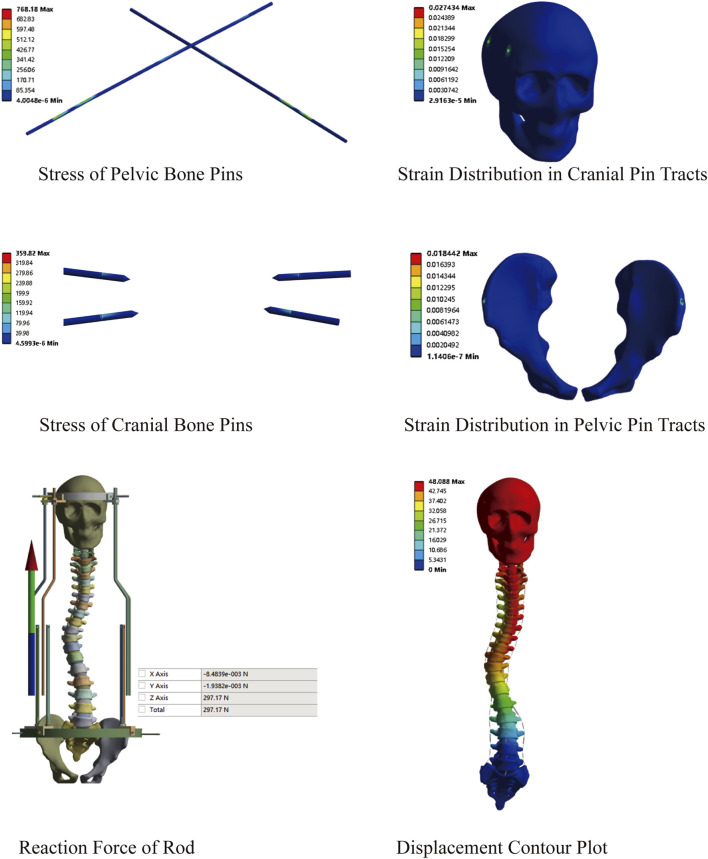
Preliminary stress analysis of the MS model under 50 mm distraction.

This model comprised a total of 3,292,529 nodes and 1,903,877 elements. First, a preliminary study on traction was conducted using the constructed MS model. An initial traction displacement of 50 mm was set, with the maximum traction force calculated based on the patient’s body weight being 297.17 N. The results showed that the overall halo strentch distance was 48.09 mm, and the reaction force of the halo-pelvics ring support rod was 297.17 N. The maximum von Mises stress of the pelvic bone pin is 768.18 MPa, and the maximum von Mises stress of the cranial bone pin is 359.82 Mpa. Given that the yield strength of 316L stainless steel is 690 MPa, the pelvic pins exceeded this limit, resulting in plastic deformation and failure. The maximum equivalent strain in the pelvic pin tract is 1.84%, while that in the cranial pin tract is 2.74%. According to Perren’s strain theory, the normal strain threshold for intact bone is 2%, indicating that during Halo frame distraction, the cranial bone has a high risk of fracture, which may lead to pin displacement (Figure 7).

### 3.3 Preliminary study on traction in SS model

**FIGURE 8:**
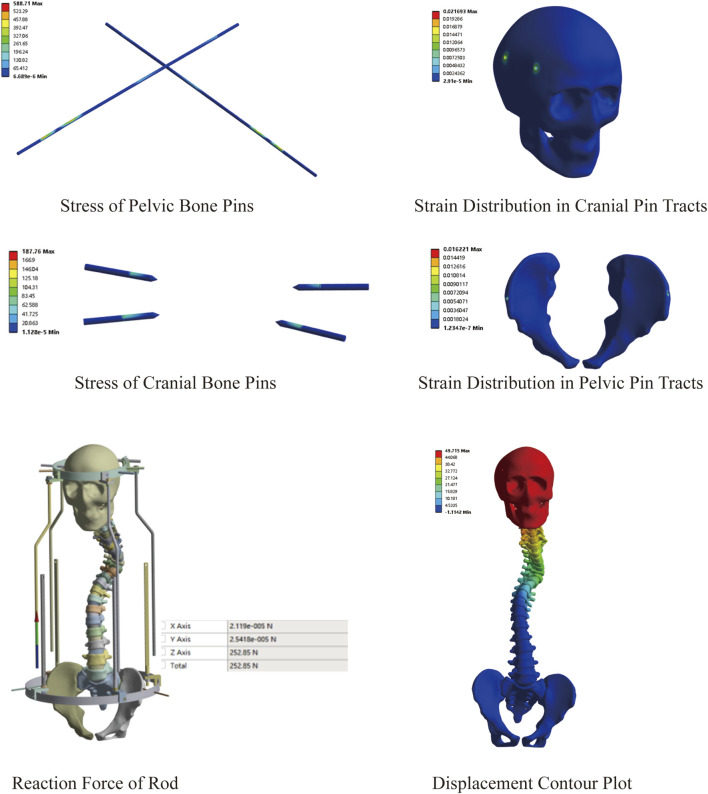
Preliminary stress analysis of the SS model under 50 mm distraction.

This model comprised a total of 2,996,116 nodes and 1,726,042 elements. Similarly, we explored the feasibility of halo-pelvic traction for SS model. Based on the patient’s body weight of approximately 42 kg and accounting for additional cervical vertebrae loading, a 230 N force was applied to the cranial region, while a downward concentrated force of 252.85 N was applied to the pelvic ring support rod, as determined by preliminary calculations. The finite element analysis revealed that the overall halo-pelvic distraction distance was 49.72 mm. The maximum von Mises stress of the pelvic bone pin is 577.71 MPa, and that of the cranial bone pin is 187.76 MPa, both of which are less than the yield strength of 316L stainless steel, which is 690 MPa. The maximum equivalent strain of the pelvic pin tract is 1.62%, and that of the cranial bone pin tract is 2.17%. According to Perren’s strain theory, the normal strain threshold for intact bone is 2%, indicating that during halo-pelvic traction, the cranial bone remains at a high risk of fracture, potentially leading to pin displacement (Figure 8). Compared to the MS model, the SS model demonstrated relatively safer traction performance. However, dynamic adjustment of traction forces remains necessary to mitigate risks.

### 3.4 Mechanical changes at different distraction distances

**FIGURE 9:**
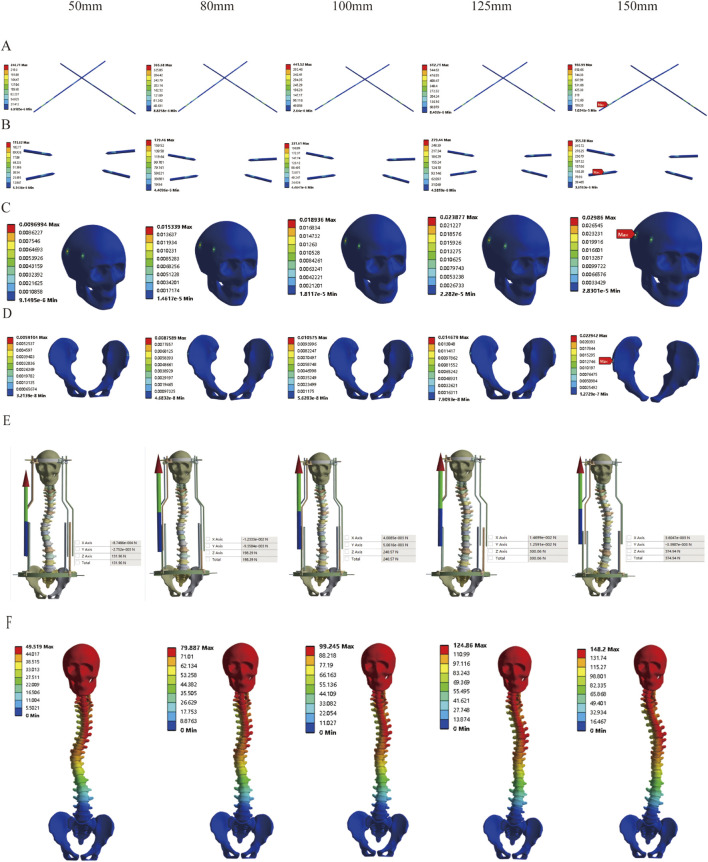
Stress variation under different distraction distances of MS model; **(A)** Stress of Pelvic Bone Pins; **(B)** Stress of Cranial Bone Pins; **(C)** Strain Distribution in Cranial Pin Tracts; **(D)** Strain Distribution in Pelvic Pin Tracts; **(E)** Reaction Force of Rod; **(F)** Displacement Contour Plot.

Based on the aforementioned findings, we further investigated the safety of dynamic halo-pelvic traction using the maximum traction force calculated for the patient’s body weight. The results of MS model demonstrated that as traction displacement increased, the stresses on both the pelvic and cranial pins progressively rose. Notably, the stress on the pelvic pins exceeded the yield strength of 316L stainless steel (690 MPa) at a traction displacement of 150 mm (Figures 9A,B). Similarly, the strain at both the pelvic and cranial pin tracts exhibited an increasing trend with greater distraction distances. Specifically, the pelvic pin tract strain reached 2.29% at 150 mm of traction, while the cranial pin tract strain reached 2.39% at 125 mm—both exceeding the 2% threshold for normal strain in intact bone as per Perren’s strain theory (Figures 9C,D). Concurrently, the reaction force of the halo-pelvic support rod followed the same trend, surpassing the applied concentrated force of 297.17 N from the pelvic ring frame when the distraction distance reached 125 mm (300.06 N), (Figures 9E,F). These findings suggest that for MS model, a distraction distance of less than 125 mm may represent a safer operational range to mitigate risks of mechanical failure and bone damage.

**FIGURE 10:**
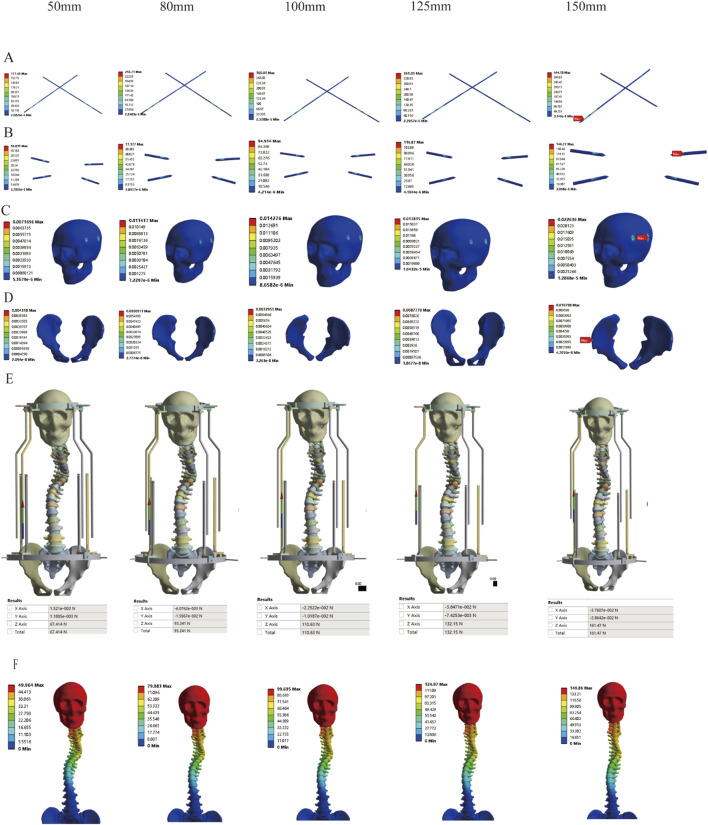
Stress variation under different distraction distances of SS model; **(A)** Stress of Pelvic Bone Pins; **(B)** Stress of Cranial Bone Pins; **(C)** Strain Distribution in Cranial Pin Tracts; **(D)** Strain Distribution in Pelvic Pin Tracts; **(E)** Reaction Force of Rod; **(F)** Displacement Contour Plot.

Correspondingly, based on previous research findings, we found that the Halo pelvics traction is suitable for SS model. However, the optimal amount of traction remains unclear. Therefore, we analyzed the mechanical changes of the Halo pelvics traction under different traction conditions. The results revealed that as the traction force increased, the stress on both the pelvic pin and the cranial pin showed an increasing trend. When the distraction distance reached 150 mm, the stress peaked at 444.18 Mpa and 146.77 Mpa, respectively, both of which were below the yield strength of 316L stainless steel at 690 Mpa (Figures 10A,B). Similarly, the strain on both the pelvic pin tract and the cranial pin tract exhibited an increasing trend with the augmentation of distraction distance. The strain on the pelvic pin tract remained below 2% of the normal bone strain level throughout the traction process. However, the strain on the cranial pin tract was less than 2% of the normal bone strain level when the traction force ranged from 50 mm to 125 mm, whereas at a traction force of 150 mm, the strain on the pelvic pin tract reached 2.26%, indicating a risk of cranial fracture and pin displacement (Figures 10C,D). The reaction force of the halo pelvics support rod also increases with the increase in distraction amount, with a maximum support rod reaction force of 161.47 N, which is lower than the concentrated force of 252.85 N applied by the pelvic ring, indicating minor risk of bone-screw interface failure at a traction force of 150 mm (Figure 10E). The displacement cloud diagrams at different distraction distances are shown in Figure 10F.

## 4 Discussion

Scoliosis involves a three-dimensional deformity of the spine, particularly severe spinal deformities that result in reduced thoracic and abdominal cavity volumes, organ compression, accompanied by cardiopulmonary insufficiency and poor nutritional status due to structural abnormalities such as shortened spinal length, vertebral rotation and displacement, and thoracic collapse. If performed as a single-stage procedure, this approach presents considerable surgical challenges including demanding correction requirements, prolonged operative duration, significant intraoperative blood loss, and elevated perioperative complication rates (Lewis et al., 2015; Bjerke et al., 2017; Riley et al., 2018). In recent years, traction technology has demonstrated significant application value in the treatment of severe spinal deformities. By longitudinally stretching the spine, it gradually relaxes the contracted tissues on the concave side, opens up the small joint spaces, intervertebral spaces, and laminar spaces, reduces the stiffness of the spine, and increases its flexibility. This effectively improves the patient’s cardiopulmonary function, enhances nutritional status, lowers the difficulty and corrective pressure of secondary orthopedic surgery, and reduces perioperative complications (Chan et al., 2016; Iyer et al., 2019a). Current clinical practice employs several traction modalities, with halo-femoral traction, halo gravity traction, and halo-pelvic traction being the most prevalent. Among these, halo-femoral traction represents one of the earliest and most widely utilized techniques. However, this method necessitates complete bed confinement, resulting in significant mobility restrictions and ambulatory impairment. The prolonged immobilization associated with this technique elevates the risk of multiple complications, including respiratory infections, urinary tract infections, and pressure ulcer development (Keeler et al., 2010). In addition, the halo-femoral traction also suffers from insufficient traction force and low orthopedic efficiency (Erdem et al., 2018). Halo gravity traction employs the patient’s own body weight as a counter-traction force to facilitate deformity correction. This technique offers distinct advantages, including the elimination of prolonged bed rest and avoidance of ring-pin fixation systems, thereby minimizing pin tract-related complications. However, its clinical utility is constrained by relatively low traction force and suboptimal correction efficienc (Watanabe et al., 2010). Halo-pelvic traction works by gradually lengthening support rods through nut rotation. With fixed pelvic and cranial rings, rod deformation generates counterforces that displace the cranial ring longitudinally, transmitting controlled traction to the spine via fixation pins. This adjustable mechanism delivers precise, continuous distraction forces (Zeng et al., 2015; Cobanoglu et al., 2018). Halo-pelvic traction offers superior patient compliance without requiring bed rest, making it ideal for severe spinal deformities. While effective for deformity correction and surgical preparation, its long-term use carries risks including pin-site infections, implant loosening, neurovascular injuries, and bone density loss (Huang et al., 2023).

It is well known that appropriate traction force and distraction distance are significantly correlated with the traction effect. Numerous finite element studies have demonstrated the efficacy of growth rod distraction in the treatment of early-onset scoliosis (Lemans et al., 2022; Pei et al., 2022). Although some studies have explored the traction force and mechanical changes in the treatment of scoliosis with halo-pelvic traction, the finite element models they constructed only applied additional loads to the scoliosis model for simulation, without considering the role of the complete halo-pelvic structure in the simulation. Therefore, these studies have certain limitations (Pei et al., 2022; Fu et al., 2024; Li et al., 2024). Therefore, this study constructed a finite element model of scoliosis deformity with complete halo-pelvic traction. Currently, there is a lack of unified standards for the validation of finite element models of scoliosis. Some scholars recommend the use of geometric morphology verification (Wang et al., 2024), which validates the effectiveness of the model by comparing the Cobb of scoliosis and vertebral offset between the measurement finite element model and X-rays. In this study, the difference between the two was minimal, thus the model is preliminarily considered to be valid. However, the biomechanical behavior of the spine is a dynamic process, and verification based solely on static geometric parameters may not fully reflect the accuracy of the model under dynamic conditions. Static or kinetic studies on cadaveric specimens are considered the gold standard. However, due to the variations in the study segments, loads, and boundary conditions adopted by different experimenters, it is challenging to obtain biological research on cadaveric specimens under identical conditions. Therefore, by comparing with the results of previous scholars, a small difference can be deemed as an indication of model validity. The T1-T4 segments exhibited reduced range of motion compared to literature values (Busscher et al., 2009; Xin, 2017), consistent with their role as the primary curve. Complementary measurements of T12-L2 stiffness and L4-L5 displacement under vertical loads showed minimal variation from published data (Brown et al., 1957; Markolf, 1972; Panjabi et al., 1977; Nachemson et al., 1979). This demonstrates that the constructed scoliosis model is effective and can be used for research. In the base model that has been successfully validated for effectiveness, a halo-pelvic ring structure simulating the traction of the halo-pelvic traction was also added, which provides a new reference for the mechanical study of scoliosis traction using the halo-pelvic ring.

In clinical practice, halo-pelvic traction is commonly used for deformities with large Cobb angles and poor spinal flexibility, such as congenital scoliosis, neuromuscular scoliosis, and tuberculous kyphosis (Muheremu et al., 2017; Xu et al., 2020; Chung et al., 2021). For mild spinal deformities, brace treatment is the preferred option (Richards et al., 2005). Deformities of ≥45° are typically treated with surgery. However, for some progressive cases, one-stage corrective surgery may increase the risk of postoperative decompensation (Cheung et al., 2019), making halo-pelvic traction as a transitional treatment. Clinical studies have shown that traction demonstrates favorable corrective capabilities and improves baseline conditions in the treatment of severe spinal deformities (Iyer et al., 2019b; Zhang et al., 2020). However, the halo-pelvic traction is rarely used for spinal deformities with minor scoliosis, especially as there is a lack of research in this direction regarding whether halo-pelvic traction can serve as a transitional treatment option for mild to moderate deformities. This study conducted a preliminary exploration by establishing a MS model. This study conducted preliminary investigations using a mild scoliosis model. The results demonstrated that applying 50 mm of traction with force calculated based on body weight significantly increased stress on pelvic pins and strain in cranial pin tracts, suggesting potential traction failure risks. This phenomenon may be attributed to excessive traction force. Li et al. (2024) conducted a finite element simulation on a patient with a 60-degree scoliosis, and their research results indicated that a traction amount of 15–20 mm is the optimal orthopedic choice. Fu et al. (2024) conducted a finite element simulation of the mechanical changes in a 61° Lenke 3 scoliosis patient undergoing halo traction, demonstrating that appropriate traction force influences the corrective outcome. Furthermore, by progressively reducing traction force through incremental traction displacements, we identified 125 mm as the critical threshold for the MS model. Exceeding this displacement not only increases pin stresses and tract strains, but may also lead to spinal morphological changes due to over-distraction (Figure 9E). This occurs because highly flexible scoliotic spines exhibit greater intervertebral mobility. During initial traction, the spine deforms readily, achieving significant correction with minimal displacement and low traction force. As traction increases, the spine approaches its flexibility limit (near-rigid state), requiring greater force to overcome rising soft tissue resistance and facet joint contact pressures. Therefore, halo-pelvic traction can serve as an interim treatment for such deformities, but it is crucial to emphasize individualized therapy and to appropriately manage the traction volume and force.

Preliminary investigation of severe scoliosis deformity with 50 mm traction displacement demonstrated that pin stresses, support rod reaction forces, and pelvic pin tract strains all remained below critical thresholds. Only cranial pin tract strain was observed, indicating that halo-pelvic traction demonstrates better applicability and safety for the SS model. Clinical evidence demonstrates that halo-pelvic traction achieves an average height gain of approximately 12 cm in severe scoliosis patients (Sun et al., 2023). While this corrective height increase does not directly correlate with traction force magnitude, it provides valuable guidance for clinical traction protocol selection. By establishing a series of traction distance, it was found that as the traction force increased, the stress on the pelvic pins, cranial pins, and the halo-pelvis ring support rods all showed an increasing trend. The stress reached its maximum at a traction distance of 150 mm, but none of them reached their ultimate values (The 316L stainless steel yield strength of 690 MPa). Similarly, the cranial pin tract and pelvic pin tract also exhibit the same trend of change, but the strain in the cranial pin tract exceeds 2% when the traction amount is 150 mm, indicating a risk of pin tract strain and cranial fracture. The distraction distance and distraction force have a significant correlation with the Cobb of scoliosis, and the Cobb decreases as the distraction distance increases (Pei et al., 2022). Halo-pelvic traction is a safe method when distraction distance is properly selected based on Cobb and deformity severity, with <150 mm recommended for severe scoliosis cases. Although this study simulated traction forces based on patient body weight to maximize realism, significant individual variations may lead to diverse biomechanical responses and treatment outcomes. For instance, patients with poor bone quality may exhibit higher stress at the pin-bone interface, increasing the risk of pin loosening or failure. Patients with well-developed muscles might require higher traction forces, whereas those with ligamentous laxity may need lower forces. Similarly, rigid spinal deformities could necessitate greater traction to achieve correction. In clinical practice, exploratory adjustment of traction force is often required for individualized treatment to enhance both corrective efficacy and safety. Further research is needed to establish optimal, patient-specific criteria for traction force selection.

This study successfully established a three-dimensional finite element model for scoliosis treated with halo-pelvic traction. The simulation results are more realistic, addressing the limitations of previous studies that directly applied stress loading for simulation, providing a methodological option for future research in this field. However, this study still has some limitations. Firstly, the finite element model established in this study did not take into account the influence of the thorax, muscles, ligaments, and spinal cord. Longitudinal traction of muscles and ligaments would impose additional stress on the spine and spinal cord. And, this study is a finite element analysis of a single individual, and there may be individual variations and applicability to different types of scoliosis in the treatment with halo-pelvic traction. Future research should increase the sample size to analyze the impact of individual differences and types of scoliosis in halo-pelvic traction. Furthermore, this study primarily focuses on macroscopic biomechanical analysis and does not include experimental validation of the deeper biological mechanisms through which traction forces affect bone and soft tissue interactions. Notably, halo-pelvic traction is inherently a dynamic process, whereas this study employed a static mechanical model to simulate the mechanical changes of the halo-pelvic structure at different distraction amounts. Future research should still focus on developing dynamic traction models to more accurately simulate the actual mechanical changes.

## 5 Conclusions

In summary, this study successfully established a finite element model for halo-pelvic traction in scoliosis, providing a methodological reference for future research in this yield. Additionally, our study found that halo-pelvic traction can be used as a transitional treatment for minor deformities, but the traction force must be appropriately controlled. For severe deformities, halo-pelvic traction is a preferable treatment option, and maintaining a distraction distance of less than 150 mm may help avoid the risk of bone-screw interface failure.

## Data Availability

The original contributions presented in the study are included in the article/supplementary material, further inquiries can be directed to the corresponding authors.
